# Supplementary material for: A field test of the dilution effect hypothesis in four avian multi-host pathogens
Source: PLoS Pathog. 2021 Jun 23;17(6):e1009637. doi: 10.1371/journal.ppat.1009637 (PMC8221496; doi:10.1371/journal.ppat.1009637)
Supplement: S1 Table — Significant relationships (p ≤ 0.05) are highlighted in bold; conditional and marginal (in brackets) R2 variance are shown. (PDF) [file ppat.1009637.s001.pdf]

Table S1. Results of the GLMMs testing the relationships between the prevalence of avian malaria *Plasmodium*, the related *Haemoproteus* and *Leucocytozoon* parasites (N=2,588), and the and the seroprevalence of WNV (N=2,544), and individual characteristics of house sparrows (age, sex, and month of capture), avian and mammal species density, richness (estimated as the raw number of different avian or mammal species registered at each sampling site), and diversity (calculated as evenness index). Significant relationships ($p \leq 0.05$) are highlighted in bold; conditional and marginal (in brackets) R^2 variance are shown.

	Plasmodium				***Haemoproteus***				***Leucocytozoon***				**West Nile virus**			
**Independent variable**	**Estimate (±S.E.)**	***χ2***	**d.f.**	***p***	**Estimate (±S.E.)**	***χ2***	**d.f.**	***p***	**Estimate (±S.E.)**	***χ2***	**d.f.**	***p***	**Estimate (±S.E.)**	***χ2***	**d.f.**	***p***
Intercept	0.42 (0.99)	0.18	1	0.67	-0.41 (1.48)	0.08	1	0.78	-1.64 (1.20)	1.86	1	0.17	0.33 (5.16)	0.01	1	0.95
Month	-0.13 (0.06)	4.35	1	**0.04**	-0.17 (0.10)	2.86	1	0.09	-0.11 (0.07)	2.21	1	0.14	-0.74 (0.31)	5.67	1	**0.02**
Sex: male	0.00[a]	0.20	1	0.65	0.00[a]	5.17	1	**0.02**	0.00[a]	1.33	1	0.25	0.00[a]	0.31	1	0.58
Sex: female	0.04 (0.09)				-0.32 (0.14)				-0.12 (0.10)				-0.23 (0.42)			
Age: unknown	0.00[a]				0.00[a]				0.00[a]				0.00[a]			
Age: juvenile	-0.17 (0.15)	5.75	2	0.06	-0.72 (0.29)	21.60	2	**<0.001**	0.06 (0.18)	37.09	2	**<0.001**	-0.37 (1.13)	1.57	2	0.45
Age: adult	-0.44 (0.20)				-0.03 (0.33)				0.87 (0.22)				0.19 (1.19)			
Avian density	-0.01 (0.01)	2.41	1	0.12	-0.01 (0.01)	1.36	1	0.24	-0.01 (0.01)	1.21	1	0.27	-0.01 (0.02)	0.32	1	0.57
Avian richness	0.01 (0.01)	0.00	1	0.96	0.06 (0.02)	9.62	1	**<0.001**	0.03 (0.02)	4.05	1	**0.04**	0.16 (0.06)	7.84	1	**0.01**
Avian diversity	0.56 (0.94)	0.35	1	0.55	-1.15 (1.38)	0.70	1	0.40	0.90 (1.10)	0.68	1	0.41	-2.80 (5.27)	0.28	1	0.60
Mammal density	-0.01 (0.02)	0.80	1	0.37	-0.05 (0.02)	7.23	1	**0.01**	0.06 (0.02)	10.20	1	**<0.001**	-0.01 (0.06)	0.05	1	0.82
Mammal richness	0.10 (0.11)	0.87	1	0.35	-0.05 (0.14)	0.13	1	0.72	-0.11 (0.15)	0.55	1	0.46	-1.16 (0.42)	7.58	1	**0.01**
Mammal diversity	-0.95 (0.49)	3.80	1	**0.05**	0.61 (0.53)	1.34	1	0.25	0.17 (0.54)	0.10	1	0.75	4.61 (1.62)	8.15	1	**<0.001**
R^2 **(%)**	15.07 (3.22)				38.18 (9.61)				37.22 (7.69)				65.36 (39.85)			

[a] Reference category.
