# Supplementary material for: A field test of the dilution effect hypothesis in four avian multi-host pathogens
Source: PLoS Pathog. 2021 Jun 23;17(6):e1009637. doi: 10.1371/journal.ppat.1009637 (PMC8221496; doi:10.1371/journal.ppat.1009637)
Supplement: S4 Table — Significant relationships (p ≤ 0.05) are highlighted in bold. Conditional and marginal (in brackets) R2 variance are shown. (PDF) [file ppat.1009637.s004.pdf]

**Table S4.** Results of the GLMMs analysing the relationship between the prevalence of the two mosquito-borne pathogens studied: avian malaria *Plasmodium* (N=2,588) and seroprevalence of WNV (N=2,544), and the individual characteristics of the house sparrows (age, sex and month of capture), avian species density, richness (measured from the raw number of different avian species registered at each sampling site) and diversity (calculated as the avian phylogenetic diversity), mammal species density, richness (measured from the raw number of different mammal species registered at each sampling site) and diversity (calculated as evenness index), and vector species richness (measured from the raw number of different mosquito species captured at each sampling) and diversity (calculated as evenness index). Significant relationships ($p \leq 0.05$) are highlighted in bold; conditional and marginal (in brackets) $R^2$ variance are shown.

| | ***Plasmodium*** | | | | **West Nile virus** | | | |
|---|---|---|---|---|---|---|---|---|
| **Independent variable** | **Estimate (±S.E.)** | **$\chi2$** | **d.f.** | ***p*** | **Estimate (±S.E.)** | **$\chi2$** | **d.f.** | ***p*** |
| Intercept | 0.99 (0.85) | 1.36 | 1 | 0.24 | -3.96 (4.26) | 0.86 | 1 | 0.35 |
| Month | **-0.13 (0.06)** | **4.25** | **1** | **0.04** | **-0.75 (0.31)** | **5.76** | **1** | **0.02** |
| Sex: male | 0.00[a] | 0.22 | 1 | 0.64 | 0.00[a] | 0.33 | 1 | 0.56 |
| Sex: female | 0.04 (0.09) | | | | -0.24 (0.42) | | | |
| Age: unknown | 0.00[a] | | | | 0.00[a] | | | |
| Age: juvenile | -0.17 (0.15) | 5.70 | 2 | 0.06 | -0.54 (1.13) | 1.40 | 2 | 0.49 |
| Age: adult | -0.44 (0.2) | | | | -0.03 (1.19) | | | |
| Avian density | -0.01 (0.00) | 3.01 | 1 | 0.08 | 0.00 (0.01) | 0.05 | 1 | 0.83 |
| Avian richness | 0.01 (0.02) | 0.48 | 1 | 0.49 | 0.17 (0.09) | 3.52 | 1 | 0.06 |
| Avian phylogenetic diversity | -0.01 (0.00) | 1.39 | 1 | 0.24 | -0.02 (0.02) | 1.05 | 1 | 0.31 |
| Mammal density | -0.02 (0.02) | 1.62 | 1 | 0.20 | -0.04 (0.06) | 0.42 | 1 | 0.52 |
| Mammal richness | 0.04 (0.11) | 0.11 | 1 | 0.74 | -0.85 (0.61) | 1.95 | 1 | 0.16 |
| Mammal diversity | -0.51 (0.52) | 0.09 | 1 | 0.33 | 3.90 (2.23) | 3.08 | 1 | 0.08 |
| Mosquito richness | 0.07 (0.06) | 1.35 | 1 | 0.25 | 0.34 (0.26) | 1.78 | 1 | 0.18 |
| Mosquito diversity | -0.72 (0.8) | 0.81 | 1 | 0.37 | 3.11 (2.88) | 1.17 | 1 | 0.28 |
| ***$R^2$* (%)** | 3.86 (14.36) | | | | 44.88 (63.10) | | | |

[a] Reference category
