# Supplementary material for: A field test of the dilution effect hypothesis in four avian multi-host pathogens
Source: PLoS Pathog. 2021 Jun 23;17(6):e1009637. doi: 10.1371/journal.ppat.1009637 (PMC8221496; doi:10.1371/journal.ppat.1009637)
Supplement: S1 Text — (PDF) [file ppat.1009637.s006.pdf]

Supporting information: methods and results

Vector sampling and community abundance and composition

Mosquitoes were captured using 135 traps (3 traps x 45 localities) with a total trapping effort of 810 trap-nights. In each trapping session, every 45 days, three traps were operated for 24 hours in all three localities of the same triplet.

Overall, 340,829 female mosquitoes belonging to 13 different species and five genera were collected. Between two and 10 species of mosquitoes were captured at each locality (mean: seven species); the species richness estimated from the rarefaction curves was in the range 1.99–6.93 (mean 4.54). Detailed information on mosquito community abundance and composition has been published by Ferraguti et al. [1]. Briefly, the most abundant species trapped were *Culex theileri* Theobald (282,891 ind.), *Ochlerotatus caspius* Pallas (21,155) and *Culex pipiens* Linnaeus (19,268), followed by *Culex perexiguus* Theobald (5,939), *Anopheles atroparvus* Van Thiel (5,387), *Anopheles atroparvus* Van Thiel (3,207), *Culiseta annulata* Schrank (2,514), *Ochlerotatus detritus* Haliday (1,495), *Culex modestus* Ficalbi (1,237), *Culiseta longiareolata* Marcquart (476), *Anopheles algeriensis* Theobald (41), *Ochlerotatus berlandi* Seguy (22), *Culiseta subochrea* Edwards (13) and *Urotaenia unguiculata* Edwards (6).

Vertebrate censuses and community abundance and composition

Five point-counts in the vicinity of the mosquito trap sampling localities were used to estimate avian density [2]. During six minutes at each sampling point, a trained observer recorded the species and their distance from the observer of all the birds seen or heard within a radius of 100 m. The density of each species at each locality was estimated using the program DISTANCE [3]. For mammal census, the number of pellets/scats was recorded along five transects (200-m long x 1-m wide) around the mosquito traps. The mammal species recorded were the common hare, rabbit, horse, cattle, red deer, fallow deer, wild boar, cat, dog and fox. Published information on the defecation rates of each species was used to estimate species density (see Supporting Information in Ferraguti et al. [4] for further details).

The reports from the vertebrate censuses indicated the presence of 143 avian and 18 mammal species in the study areas, with a mean of 16 bird species (range: 2–33) and two mammal species (range: 0–7) for each locality. Mean rarefaction values were 13.05 for avian (range: 6.96–24.99) and 1.53 for mammal (range: 0–3.96) communities. The mean

evenness values calculated for each locality were 0.67 for avian (range: 0.28–0.94) and 0.32 for mammal (range: 0–0.95) communities. For the avian community, the mean avian phylogenetic diversity per locality was 99.12 (range: 40.15–190.24). Detailed information on vertebrate community abundance and composition has been previously published by Ferraguti et al. [4].

1. Ferraguti M, Martínez-De La Puente J, Roiz D, Ruiz S, Soriguer R, Figuerola J. Effects of landscape anthropization on mosquito community composition and abundance. Sci Rep. 2016;6: 29002. doi:10.1038/srep29002

2. Buckland ST. Point-transect surveys for songbirds: robust methodologies. Auk. 2006;123: 345–357.

3. Thomas L, Laake JL, Strindberg S, Marques FFC, Buckland ST. Distance 5.0. release “x” 1. UK: research unit for wildlife population assessment. University of St. Andrews, UK; 2006.

4. Ferraguti M, Martínez-de la Puente J, Bensch S, Roiz D, Ruiz S, Viana DS, et al. Ecological determinants of avian malaria infections: an integrative analysis at landscape, mosquito and vertebrate community levels. J Anim Ecol. 2018;87: 727–740. doi:10.1111/1365-2656.12805
