# Supplementary material for: A field test of the dilution effect hypothesis in four avian multi-host pathogens
Source: PLoS Pathog. 2021 Jun 23;17(6):e1009637. doi: 10.1371/journal.ppat.1009637 (PMC8221496; doi:10.1371/journal.ppat.1009637)
Supplement: Attachment — Submitted filename: Response to Reviewers.docx [file ppat.1009637.s007.docx]

**Comments to Reviewers:**

**Part I – Summary**

Please use this section to discuss strengths/weaknesses of study, novelty/significance, general execution and scholarship.

Reviewer #1: The manuscript brings together a number of interesting components of the dilution effect hypothesis (DEH) and is a timely and methodologically sound contribution. The prevalence of four pathogens estimated from avian species, is regressed against a number of explanatory variables that are expected to reveal a mechanistic understanding of the DEH. Prevalence in avian species is related to the richness, diversity, density, phylogenetic diversity, and evenness of host and vector species assemblage. The results showed a number of inconsistencies with key assumptions of the dilution effect hypothesis. The key finding was that the prevalence of most of the avian pathogens studied, exhibited a positive relationship with community parameters used commonly in DEH research. The study is novel and of broad interest because empirical data derived from a tractable study system is used to get at the mechanics of the relationship between biodiversity and disease risk.

Reviewer #2: I have reviewed a previous version of this manuscript. The authors have adequately addressed my comments and the manuscript has been substantially improved. I have no further comments.

**Part II – Major Issues: Key Experiments Required for Acceptance**

Please use this section to detail the key new experiments or modifications of existing experiments that should be absolutely required to validate study conclusions.
Generally, there should be no more than 3 such required experiments or major modifications for a "Major Revision" recommendation. If more than 3 experiments are necessary to validate the study conclusions, then you are encouraged to recommend "Reject".

Reviewer #1: The manuscript covers a lot of ground by introducing many concepts developed by others, to explain mechanics of the DEH. At times, the reading was difficult work for me, and also likely for the uninitiated virologist does not savvy in the terminology of community ecology. This comes about primarily due to the vagueness or incomplete explanation for key concepts introduced at the beginning of the manuscript. These issues can be rectified in a rewrite that provides specific references (e.g., point to, and give details of, the particular key finding of interest presented in a given reference), and explains more thoroughly the cited work already given. In particular, I found the premise of, and explanation for, additive and subtractive assembly incomplete, and difficult to integrate with the other concepts presented in the introduction, results, and discussion. These are major concerns. Specifics of minor concerns are given below.

Reviewer #2: (No Response)

We have revised the manuscript and in particular the introduction to make it clear from the start the definition of the main terms used throughout the paper. We have further clarified the issue of additive and subtractive assembly because we now realize that our arguments in that section were not sufficient.

**Part III – Minor Issues: Editorial and Data Presentation Modifications**
Please use this section for editorial suggestions as well as relatively minor modifications of existing data that would enhance clarity.

Reviewer #1: Line 31: Suggestion. A succinct statement delineating the relevance of density- and frequency-dependent transmission in relation to pathogen-host encounters and the dilution effect hypotheses, would provide a general basis for developing the subsequent arguments that rely on variation in species density.
We are a bit confused here because dilution is predicted both for density- and frequency-transmitted pathogens and consequently it does not seem relevant to introduce these concepts into the Abstract of the manuscript. In addition, we are dealing with vector-borne pathogens, where both vectors and hosts are relevant. However, we have slightly modified this part. Please see new lines 34-36.

Line 49: ‘possessing’.

Changed.

Line 52: Be specific when describing variables of relationships that were found to important. What parts of ‘biodiversity’ were regressed here. As the strength of the manuscript is its mechanistic perspective on the DEH, a conclusion commenting on this theme would be good, i.e., how did community composition, species identity, and abundance influence prevalence in avian hosts.

Done, see lines 53-58.

Line 89: Expression; “…reported in the wild”, or, have been observed in wild populations?

The sentence has been modified.

Line 96. “proposed by the dilution effect”, or, in the DEH…

Changed.

Line 110: Non-experts may not know what community composition refers to, and how it differs from diversity (and structure). For example, composition typically separates the species identity and species abundance components of diversity.

The community composition concept has been clarified by adding the importance of species abundance, as well as community composition, and by describing the main findings of the cited studies.

Lines 117 to 128: I read this part several times, and the connection between the hypotheses of Rohr et al. (2020), and the analyses conducted in the manuscript are unclear. Rohr et al. (2020) states "When community (dis) assembly is substitutive, amplification can occur when the addition of individuals of new, competent host species reduce the density of less competent host species. Amplification or dilution can occur when competent hosts or non-competent hosts, respectively, are added to or subtracted from communities via the sampling effect (that is, more diverse communities are more likely to contain a host species that either strongly increases or decreases disease)". The authors provide little information (and no empirical data that I can see) on whether the addition of non-competent or competent hosts occurs in the study system.

We are sorry for this misunderstanding. We were not testing Rohr’s hypothesis regarding the addition of competent or non-competent species. Unfortunately, species specific information on competence for the four studied pathogens is not available for any avian community in the world. What we were testing in our study was whether species-rich communities had higher densities of vertebrates or vectors than species-poor communities, and consequently whether or not they fit a substitutive or additive assembly model. We have clarified this point in the new version of the manuscript. See lines 144-149.

Lines 145 to 142: This statement is not entirely correct. The Roche et al. (2013) model assumed that the most abundant vector and reservoir species had the highest susceptibility (i.e., not competence). In their model, competence was "modulated" (i.e., correlated) by susceptibility. The difference between susceptibility and competence is not trivial and the differences deserve attention because it may alter how the conclusions of the study are interpreted.

We have clarified this difference in the text.

Line 149: The authors introduce the term ‘assembly’ that relates to successional pathways and the temporal changes in biological communities. This is clear enough, and the references to Rohr et al. (2020) set the conceptual background for expectations given either additive or subtractive assembly. However, I am not sure how these hypotheses fit with the analyses presented in the manuscript. I struggled to see a connection between the objectives of the study, broadly to look at avian prevalence as explained by the composition of the vector and host assemblages, and the hypotheses for subtractive and additive assembly. A rewrite should include a better explanation of this connection, if indeed one is intended.

We would like to respectfully disagree with this interpretation of the term ‘assembly’. It is true that assembly can imply succession if you are studying temporal changes in a community. However, the term ‘assembly’ is widely used in biogeography and community ecology to describe how species are integrated into communities (Colorado Zuluaga, 2015. How ecological communities are structured: a review on ecological assembly rules. *Revista EIA*, (24), 27-53; Pavoine & Bonsall, 2011. Measuring biodiversity to explain community assembly: a unified approach. *Biological Reviews*, 86(4), 792-812). It is important to understand how changes in species richness and vertebrate and/or vector density are related since it can explain why dilution, amplification or non-significant effects are found in different systems (see, for example, Figure 3 in Johnson et al. 2015). Despite this, we have modified the sentence in order to clarify this point; see lines 169-173.

Line 176: What are “all the factors”? Understanding “all the factors” is not likely.

We have modified the sentence as follows: “We used different metrics of species richness and diversity in vector and host communities to understand their potential effect on the occurrence of DEH”

Lines 183 to 185: I do not understand the meaning of this sentence.

We have modified this sentence to make it clear that our results are robust for a wide range of biodiversity-related metrics. It does not matter whether or not you analyse species richness, species richness corrected for sampling effort, evenness, Shannon index, or diversity corrected for phylogenetic distance. The results in our study do not change: the relationship of these parameters with the prevalence of pathogens is positive, and never negative. In a first version we analysed species richness and Shannon index and, in light of reviewers’ suggestions, we added species richness corrected for sampling effort, evenness and diversity corrected for phylogenetic distance. See new lines 206-211.

Line 186: What do the authors mean by “evenness analysis for richness and diversity parameters”? Evenness is a parameter, not a type of analysis.

The sentence has been modified to read “we report the results of the analyses using the number of species projected from a rarefaction curve to estimate richness, and evenness as a measure of diversity”. Please, see lines 209-211.

Lines 194 to 199: As I have understood the "additive" and "subtractive" hypotheses of assembly in respect to the relative proportions of competent to non-competent hosts (as presented in the background above: Rohr et al. 2020), I do not know how measuring correlations (e.g. vector richness vs. vector density) informs on the numbers of competent and non-competent hosts (or high/low susceptibility hosts), or whether this proportion, or the phenotype introduced during community assembly, is important to the direction of the manuscript. How does the correlation inform on the subtractive and additive hypotheses for assembly, and then, how do the mode(s) of assembly relate to the study system?

The aim of these analyses was to test whether or not species-rich communities hold higher densities of vertebrates and/or vectors. A key criticism of the dilution effect is that species-rich communities will hold more vertebrates and so will allow more abundant populations of vectors to develop. Here we show that in our study system this is not the case and species-rich communities do not have higher avian densities, and that mosquito abundance is not greater where there are more birds living. We have clarified this issue in the first line of the paragraph.

In the Methods, Lines 464 to 467; i.e., the mean (species?) relative (or total?) abundance of mosquitoes captured each day was calculated. How exactly does daily variation in the mean abundance inform on successional changes in the mosquito assemblage? And how does it inform on the proportions of competent (i.e., to transmit) to non-competent host phenotypes?

This was not the aim of the analyses. The aim was to determine whether or not richer mosquito communities have more mosquitoes (as assumed by Roche et al.). In the second page of their paper, they indicate that “It is important to point out that using our framework any increase or decrease in the species richness of vectors or reservoirs species will also result in a change in total number of individuals in the community”). Our results indicate that this assumption is not realistic. Still, we have added a new sentence to further clarified this issue, see lines 495-497.

The references given for the work of Rohr et al. (2020) and Randolph and Dobson (2012) should direct the reader to specific aspects of each of these studies. I read them and could not find an obvious connection. The work by Randolph and Dobson (2012) refers to theoretical (i.e., "in principle") outcomes of adding non-competent hosts to an assemblage, so the phenotype is known or assumed in this case. Given that the phenotype (i.e., non-competent) is assumed/known, decreases in encounters with other hosts [“humans”] can be expected. The absolute number of vectors is important, [“not the proportion of vectors infected”], but under the assumption that non-competent hosts have been added. Here, I am lost.

We have clarified the relationships between the references and the analyses. In particular, we indicate that Randolph and Dobson proposed that dilution was not likely to occur because higher vertebrate densities will encourage the development of higher vector densities (see, for example, the end of the second paragraph on page 849 of their paper, or the second paragraph on page 850). The case of Rohr et al. has been commented on above.

Line 200 to 204: Why are there no references to prevalence as the dependent variable? The Results section begins with an analysis of prevalence. It is not clear at this stage of the manuscript what the objectives of the study are, nor the hypotheses being tested; because there are no explicit statements to this effect.
In the new version of the manuscript we have improved our explanation and description of the hypotheses tested by stating them much more clearly in lines 195-200.

Lines 210 to 232: The data are impressive, and the motivation to analyse the composition of assemblages as well as successional pathways is a very novel aspect of the manuscript. The introduction argued that community composition (taxonomic identity and their abundances) and diversity were important to separate, in understanding disease risk. This proposition is a strong component of the manuscript, but the regressions include largely independent variables for richness and diversity. Only one model speaks to composition (i.e., evenness). The results that point to the regression modelling are clear, but I am not sure what information I am supposed to gain from the other results reported here. Are they for the purposes of understanding the assembly hypotheses? Are the results of rarefaction of secondary importance? Rarefaction is typically used to address uncertainty in sample completeness and can be compared to the values of the raw observations in a Table somewhere, usually in the Supplementary Information. If the rarefaction results and mean values are of primary importance, the information would be easier to digest if it were presented in a compact, summary form that informs on their purpose. Do the mosquito abundance results and those referenced in the Ferraguti et al. (2016, 2017) papers, comprise the main comment on community composition made in the manuscript?

Following this suggestion, we have modified this section by reducing the text and moving most of this information to the supplementary material (see Supplementary Information). The aim of this section was to introduce readers to the different mosquito species present in the area and provide some basic information about the study system. However, we would be happy to include additional changes if necessary.

In addition, we have included different variables reflecting the host communities in our statistical models in order to address comments by reviewers of previous versions of the manuscript. Thus, we ran models considering the classical metrics of host communities (richness and rarefaction curve), and also considered the identity of the hosts (evenness index) and their phylogenetic relationships (in the case of the avian host). In all these cases, similar conclusions were obtained, thereby further supporting the robustness of the results reported here. Please note that in the first evaluation the same reviewers requested the use of rarefaction for the estimation of species richness and the estimation of diversity taking into account phylogenetic diversity.

Lines 214 to 215: What does this mean: “and a rarefaction value of
4.54 (range 1.99 – 6.93), in each locality”? It reads like the same mean value was found in each locality.

Corrected to “Between two and 10 species of mosquitoes were captured at each locality (mean: seven species); the species richness estimated from the rarefaction curves was in the range 1.99–6.93 (mean 4.54).” This information is now available in the Text S1.

Line 225: Another example of poor expression: “Vertebrate counts reported the total presence of…” Vertebrate counts do not report. We are sorry for the poor phrasing here. We have clarified this sentence in the Text S1 and now state that “The reports from the vertebrate censuses indicated the…”. The English has been reviewed by a native speaker.

Line 236 and 247: Tables 1 and 2 include independent variables demographic variables (i.e., the population level), but I do not know its relevance to what has been introduced thus far. How do the demographic parameters fit into the general objectives and hypotheses?

We have now explained the demographic parameters in methods. Obviously, the age of the individuals may affect the prevalence of the parasites as juvenile birds have been exposed to vectors for less time. In the same way, females may be more exposed to mosquitoes when on the nest. Consequently, we controlled for these factors in the analyses. However, not controlling for these variables does not change the results.

Line 250: …models that included…

Changed.

Line 260: Is this a regression of species richness and total abundance (i.e., total number of mosquitoes collected) at each site? This sentence appears to contradict the former. In this section we were comparing results for mosquito total abundance with both rarefaction and with the number of mosquito species. Different results were found for each ‘richness’ parameter. We have now clarified this paragraph; see lines 252-259.

Line 261 to 262: Is “site” the same as “locality”?

Yes. We have homogenised the use of the term ‘sampling site’ with sampling locality throughout the whole manuscript.

Line 281: It would be great to have a concise summary of the main finding in respect to how mechanics related to community composition influenced prevalence in the study system: i.e., “…separate the effects of biodiversity from those due to the presence of particular host species”.

We have added a small summary of the main finding in lines 279–288.

Initially in the discussion, there are many references to the work of others, but few in respect to the findings presented in the manuscript. The main findings appear to relate to the positive relationships between avian prevalence and parameters such as richness and diversity. This type of result does not live up to the direction of the study laid out in the introduction: “pathogen prevalence may be due to either the presence of key species or to the community composition rather than to any intrinsic property of biodiversity”. From that point in the introduction, the exciting topic of community assembly is introduced, thus raising the expectation of the reader beyond the typical disease-diversity paradigm. A change of emphasis in the discussion would help point out where the manuscript contributed most strongly to the field.

We are sorry, but the aim of the paper was to test the dilution effect and some of its main assumptions. As we have commented above, detailed information regarding the host competence of most of the avian species in the area is not available and so entering into a detailed analysis of the host competence of each community lies beyond the scope of this paper.

Line 298: In its current state, the main finding appears to be: “these results support the incidence of increased pathogen transmission in areas with a richer avian community contrary to the expectations of the DEH.” With all the analyses and results given about other hosts and vectors, is there not a main finding that can be related to the composition of the various assemblages?

We did not analyse the composition. Host competence for most of the species/pathogen combinations is unknown and so there was no room for discussing the results on a species-by-species basis. In addition, this is beyond the scope of the paper, which focusses on the relationship between pathogen prevalence and the general descriptors of biodiversity

Line 313 and 314: This relationship is interesting because it relates to composition (taxonomic identity and abundance). Are there any results that support this speculation?
We have added a missing citacion to a previous work of ours that shows that *Cx. perexiguus* infected by Usutu virus is more abundant in areas with a greater richness of avian species (Roiz et al. 2019, Ecohealth, https://doi.org/10.1007/s10393-019-01441-3).

Line 314: Is “community structure” supposed to be “community composition” here? The structure of a community is influenced by abiotic variables and so does not fit the context of the sentence.

Yes, we are sorry for this misunderstanding and we have changed “structure” for “composition”.

Line 336: …than other species.

We are sorry, but we do not understand this comment.

Lines 344 to 345: Which relationship exactly is being referred to? Is it prevalence in avian species as predicted by mammal density?

We were referring to the relationship between the prevalence of *Haemoproteus* and mammal density, and the prevalence of Leucocytozoon and mammal density. We have now clarified this in lines 363-364 as follows: “However, mammal density was negatively related to Haemoproteus prevalence and positively associated to Leucocytozoon prevalence”

Lines 368 to 371: Randolph and Dobson (2012) frame infections rates in terms of vector abundance, not vector density, and host density. If vector density is not correlated with vector abundance, how is the comparison with the referenced work valid?
We have no reason to assume that the number of mosquitoes captured does not represent vector density. In fact, what is most relevant is neither vector density nor vector abundance, but their relationship to the host equivalent.

Line 381: It is not apparent to me how the study showed additive community assembly. We have reported that more species of mosquitoes are present in the areas with greater densities/abundances of mosquitoes, and this is what we would expect in an additive community assembly.

Lines 403 to 404: “pattern” or patterns; “those” or that?

The plural form has been corrected.

Line 410: analysed.

Changed.

Line 418: I did not find information about substitutive assembly in these two references.
We are sorry for this mistake and we have checked and corrected the references.

Line 536: I see no references in the introduction about why demographic variables are important predictors of risk.

Our aim here was not to test the effect of age and sex on pathogen prevalence as it has already been demonstrated in many previous studies. However, it is still necessary to control for them, and we have made this clear in the methods section. See lines 560-563.

Line 541: Richness is ordinal (unless rarefied).

Richness is a continuous variable and in the Introduction we have clarified that for this parameter we will use the rarefaction curve. Please see lines 198-200: “In particular, in the case of species richness, we estimated the rarefaction curve (for the sake of clarity, hereafter referred to as ‘richness’) [20,38] to take into account the differences between localities in the number of samples taken”. Nevertheless, we have maintained the term ‘rarefaction’ in the text to clarify the parameter analysed in specific models.

Reviewer #2: (No Response)
